# Age-Related Changes in Segmental Body Composition by Ethnicity and History of Weight Change across the Adult Lifespan

**DOI:** 10.3390/ijerph13080821

**Published:** 2016-08-13

**Authors:** Simiao Tian, Béatrice Morio, Jean-Baptiste Denis, Laurence Mioche

**Affiliations:** 1 Department of Scientific Research Project, Affiliated Zhongshan Hospital of Dalian University, 116001 Dalian, China; 2 Unité Mixte de Recherche 1397, Laboratoire Carmen, Université Lyon 1, INSERM U1060, INSA de Lyon, Universités Rockefeller et Charles Merieux Lyon-sud, 69000 Lyon, France; beatrice.morio@lyon.inra.fr; 3 Unité de Recherche Mathématiques et Informatique Appliquées, Institut National de Recherche Agronomique, F-78352 Jouy-en-Josas, France; jean-baptiste.denis@jouy.inra.fr; 4 Unité de Nutrition Humaine, Unité Mixte de Recherche 1019, Institut National de Recherche Agronomique, F-63000 Clermont-Ferrand, France; laurence.mioche63@gmail.com

**Keywords:** multivariate modelling, aging, body composition

## Abstract

This study assessed age-related changes in body composition (specifically in trunk fat and appendicular lean masses), with consideration of body mass index (BMI) at age 20 years (BMI reference age, “BMIref”), ethnicity and lifetime weight change history. A cross-sectional dual-energy X-ray absorptiometry-based dataset was extracted from the U.S. National Health and Nutrition Examination Survey (NHANES) 1999–2004. Only European-American and African-American subjects were used (2705 men, 2527 women). For each gender and ethnicity, 6 analytic cases were considered, based on three BMIref categories (normal, overweight and obese, being 22, 27 and 30 kg/m^2^, respectively) and two weight contexts (stable weight or weight gain across the lifespan). A nonparametric model was developed to investigate age-related changes in body composition. Then, parametric modelling was developed for assessing BMIref- and ethnicity-specific effects during aging. In the stable weight, both genders’ and ethnicities’ trunk fat (TF) increased gradually; body fat (BF) remained stable until 40 years and increased thereafter; trunk lean (TL) remained stable, but appendicular lean (APL) and body lean (BL) declined from 20 years. In the weight gain context, TF and BF increased at a constant rate, while APL, TL and BL increased until 40–50 years, and then declined slightly. Compared with European-American subjects of both genders, African-American subjects had lower TF and BF masses. Ethnic differences in body composition were quantified and found to remain constant across the lifespan.

## 1. Introduction

Aging is associated with substantial changes in body composition. Reductions in body lean (BL) or body fat-free (BFF) masses occurs during aging [1], together with increases in body fat (BF) related to the accumulation of adipose tissue, particularly in the abdominal region [2]. These changes are closely linked with muscle strength reduction during aging [3]. The loss of muscle mass and/or strength, known as sarcopenia, may negatively impact physical function, and lead to functional impairment and disability [4,5,6]. Meanwhile, the accumulation of BF may be associated with a number of metabolic risk factors and lead to an increased prevalence of chronic metabolic diseases [7]. Many studies have shown that increased abdominal fat mass is an independent risk factor for hypertension, stroke, and Type 2 diabetes [8,9,10]. Other reports suggest that upper body fat (truncal fat) has been strongly associated with insulin resistance, metabolic risk factors, and their disease outcomes [11,12].

Although body composition and its age-related changes have a strong genetic component [13], they are likely influenced by external factors such as social environment and physical activities [14]. Assessing these changes in segmental body composition (SBC) with aging may be important for making pre-diagnoses for the prevention of morbidity and mortality risk [15]. Most studies on age-related changes in body composition were derived from cross-sectional datasets [16,17]. One weakness of such studies is that they do not account for birth cohort effects [18,19,20]. In Ding et al.'s longitudinal study on subjects aged 70–79 years using dual-energy X-ray absorptiometry (DXA) [18], they reported that (1) at the same age, later birth cohorts had greater BF and BL masses than earlier cohorts of both genders; (2) within each cohort, BF initially increased with age and then decreased after age 80 years, while BL decreased with age, especially in men; and (3) although the amount of BF was much less than that of BL, the increase in BF was greater than of BL, which led to an increase in BF percentage. Recently, Mioche et al. [21] proposed a nonparametric model to predict SBC from easily acquired covariates. They validated their approach comparing various body composition studies and determined the influences of other variables such as ethnicity and BFF assessment methods [22]. As a result, their methodology could be adjusted to overcome some of the drawbacks of using a cross-sectional dataset for an age-related study [21].

In the present study, we were interested in the age-related changes in SBC of different ethnic and BMI contexts. Using a nonparametric model, we conducted a secular trend analysis of cross-sectional SBC data. The aims of our study were: (1) to appraise the mean age-related changes in SBC for different study cases; (2) to develop a parametric model from nonparametric models, for smooth graphical presentation and easy interpretation; and (3) to assess BMI- and ethnicity-related differences in body composition changes with aging.

## 2. Materials and Methods

### 2.1. Samples

We obtained data from the National Health and Nutrition Examination Surveys (NHANES). The NHANES is an ongoing, cross-sectional study used to assess the health and nutritional status of the civilian, non-institutionalised U.S. population, and has been conducted by the Centers for Disease Prevention and Control since 1971. It uses a stratified, multistage probability sampling design of households, thus allowing for national, population-level estimates. NHANES is collected on a continuous basis and released every 2 years with the use of standardised protocols. Participants were interviewed in their home and then invited to undergo physiologic and anthropometric examinations at a mobile examination centre (MEC). The NHANES interview included demographic, socioeconomic, dietary, and health-related questions. The physical examination consisted of medical, dental, and physiological measurements, as well as laboratory tests. All data were anonymised. To produce reliable statistics, NHANES over-samples individuals 60 years and older, African Americans, and Hispanics. All of the survey contents and procedures are available online at http://www.cdc.gov/nchs/nhanes.htm. Samples for this study were extracted from the NHANES website for the 1999–2004 period (http://www.cdc.gov/nchs/about/major/nhanes/). The NHANES dataset complies with the Declaration of Helsinki, the National Center for Health Statistics Ethics Review Board approved the protocols, and written informed consent was obtained from each participant.

Subjects were characterised by covariates, such as gender, ethnicity, age, height, weight and waist circumference. A preliminary study related to anthropometric values across the lifespan was conducted among Hispanic-American (HA), European-American (EA) and African-American (AA) subjects. The results of that study showed that within the same BMI level and age interval, HA subjects had different height, weight and waist circumference values compared to EA and AA peers. Consequently, for the present study, we only retained EA and AA subjects aged 20–85 years, with BMIs ranging from 18–40 kg/m^2^. This selection resulted in a sample size of 2705 men (1984 EA men and 721 AA men) and 2527 women (1830 EA women and 697 AA women). Height, weight and waist circumferences were considered as a similarity criterion between different subjects. Height was assumed to remain constant across the lifespan. Meanwhile, weight and waist circumference had two different contexts of change (see below). The analysis was conducted on men and women separately.

### 2.2. Segmental Body Composition

Whole-body and segmental body compositions were assessed using dual-energy X-ray absorptiometry (DXA) (Hologic QDR 4500A fan-beam densitometer for NHANES). For the NHANES dataset, detailed descriptions have been published elsewhere [23]. Briefly, whole-body DXA scans were administered in the NHANES mobile examination centre to eligible participants during the 6-year period from 1999 to 2004, and participants with certain physical conditions were excluded from the DXA examination [24]. The DXA scans permit quantification of whole-body and multiple regional components, including bone mineral content, fat and lean soft tissue. BF and BL masses, trunk fat (TF) and lean (TL) masses were thus determined [25]. The appendicular lean mass (APL) was the sum of the arm and leg lean masses [26]. In the present study, we are interested in these five components of SBC, as they are significant measures used in health assessment.

### 2.3. Analytic Cases

For each gender, we considered two ethnic groups (EA and AA), two weight contexts to mimic tow life situations (reference profile, RefProf and gain profile, GainProf) and three BMI categories (normal-weight, overweight and obese categories), making a total of 12 analytic cases.

For each BMI reference (BMIref, defined as BMI at age of 20 years), the height, weight and waist circumference profiles were the same for the two ethnicities. The starting values in men and women are given in Table 1. The RefProf assumed that height, weight and waist circumference remained constant with aging. The GainProf assumed that weight remained constant until 40 years, then increased by 5% per decade until 60 years and then stabilized. Similarly, waist circumference was assumed to remain constant until 40 years, then increased by 3 cm per decade until 60 years after which it stabilised [27,28,29].

### 2.4. Age

In the present study, we conducted both nonparametric and parametric modelling (described below). In the nonparametric modelling, age was converted into a categorical covariate and categorised into six intervals: 20–29, 30–39, 40–49, 50–59, 60–69, and ≥70 years old. A preliminary study showed that for each ethnic group, this categorization ensured adequate subset sizes for our nonparametric modelling. In contrast, for parametric modelling, age was considered as a continuous covariate.

### 2.5. Statistical Modelling and Analysis

A nonparametric approach was first used to assess segmental body composition (SBC) changes with age. This nonparametric approach followed the idea of Mioche et al. [21]. Briefly, with respect to each analytic case (each row in Table 2), our nonparametric modelling automatically selected the most similar individuals from NHANES based on their anthropometric values and ethnicity, these individuals were used to form a subset for this analytic case (with notation found in column 5 of Table 2). It then predicted SBC by averaging the values of the subset [17]. This provides our nonparametric model that is flexible and valid because the SBC changes for each age interval are derived from subjects with similar anthropometric characteristics, notably weight and waist circumference.

For smooth graphical representation, several multiple linear models were proposed to successively assess the effects of BMI category and ethnicity on age-related changes in body composition (Table 3). For the effect of BMI category, the models were tested from the simplest ($M0_{B}\left(A\right)$) to the complicated ($M3_{B}\left(A\right)$), and for the ethnic effect, the models were tested from the simplest ($M10_{B,E}\left(A\right))$ to the complicated ($M14_{B,E}\left(A\right))$. For a given gender (either men or women) and given weight change context (either RefProf or GainProf), a combined subsample was built by pooling subset_RefProf_#1,…, subset_RefProf_#6 for the RefProf context, or subset_GainProf_#1,…, subset_GainProf_#6 for the GainProf context (the notation of the combined subsample was found in column 6 of Table 2), and the models were fitted using this combined subsample. And then the standard error of the estimate (SEE) was calculated for each model using the following equation:

$$SEE=\;\sqrt{\frac{\sum_{i=1}^{n}{\left(y_{i}-{\hat{y}}_{i}\right)}^{2}}{n-p}}$$

where n is the sample size and p is the number of parameters in the model. Finally, we selected the best model by considering the trade-off between SEE and the number of model parameters.

Statistical calculations and analyses were performed using version 2.12.2 of the R software [30], a language and environment for statistical computing.

## 3. Results

### 3.1. Sample Characteristics

The mean and standard deviation for the five studied SBCs are provided in Table 4 and Table 5, for men and women, respectively. Generally, for each gender and ethnicity studied, segmental and body fat masses increased until 60 years and declined afterwards, whereas lean masses tended to remain stable until 60 years, after which they decreased gradually. More precisely, in EA men, TF and BF increased gradually until 60–69 years then declined, while TF and BF increased steadily over the lifetime of AA men. With respect to lean masses for EA men, APL, TL and BL increased until 40–49 years and then decreased. In contrast, for AA men, APL, TL and BL were stable until 60–69 years before declining. Similar results were found in EA and AA women expect that AA women’s TF and BF increased until 40–49 years, was then stable until 60–69 years, and then declined.

### 3.2. Model Selection

Table 6 shows the SEE value of nonparametric and parametric models of the effect of BMI. For the two contexts of weight trends in European-American men and women, $M3_{B}\left(A\right)$ yielded the lowest SEE values, but had the greatest number of parameters. However, in comparison with nonparametric models and other parametric models, $M1_{B}\left(A\right)$ enabled similar SEE values to $M3_{B}\left(A\right)$, and especially required fewer parameters than $M3_{B}\left(A\right)$. In addition, an ANOVA test showed that $M1_{B}\left(A\right)$ values were significantly different than $M0_{B}\left(A\right)$ values. This underlined an additive model with the covariates BMI and age. $M1_{B}\left(A\right)$ indicated that there was a BMI-related difference in SBC in each age class, but all of the BMI categories shared the same trends in aging. Thus, $M1_{B}\left(A\right)$ was retained to model age-related changes in body composition for different BMI categories.

Based on $M1_{B}\left(A\right)$, the effect of ethnicity was then examined following the same methodology. Proposed parametric models were described in Table 3. The SEE values of nonparametric and parametric models were shown for men and women in Table 7. The nonparametric model and $M14_{B,E}\left(A\right)$ yielded the lowest SEE values, but the difference was not important in comparison with $M11_{B,\;E}\left(A\right)$. In addition, an ANOVA test showed that $M11_{B,\;E}\left(A\right)$ was significantly different to $M10_{B,\;E}\left(A\right)$; therefore $M11_{B,\;E}\left(A\right)$ was retained as the model for ethnicity-, BMI- and age-related changes in body composition.

### 3.3. SBC Trends in Aging

For the sake of simplicity, only the age-related trend curves for normal weight EA subjects (reference curve) are drawn. Based on the additivity of the retained model $M11_{B,\;E}\left(A\right)$, the curves that correspond to other BMI- and ethnicity-specific subjects are simply a vertical translation of the reference curve.

**Figure 1:**
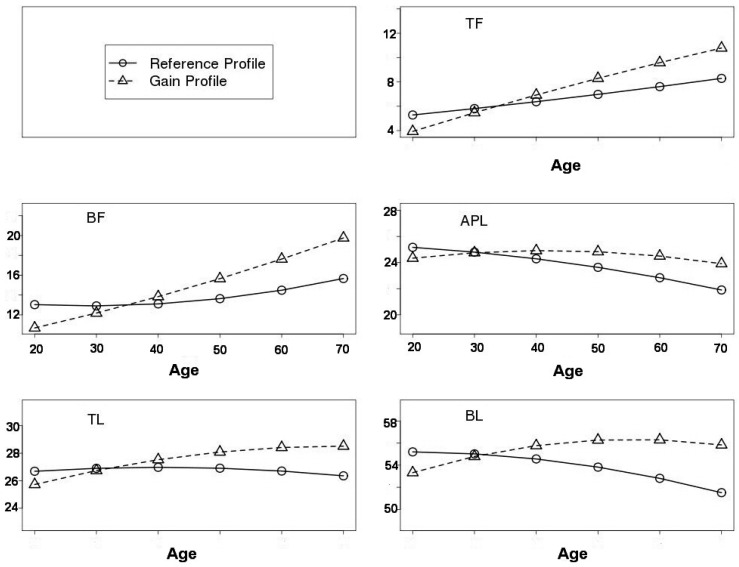
Age-related changes in segmental body composition for NHANES European-American normal men. Age is on the *x*-axis, and estimates of segmental body compositions from $M11_{B,E}\left(A\right)$ are on the *y*-axis. Reference profile is represented by (o) and gain profile by (Δ).

**Figure 2:**
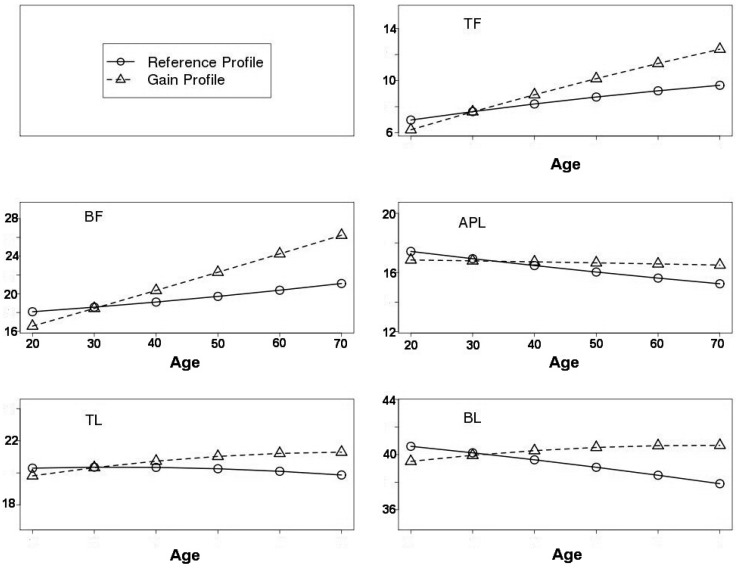
Age-related changes in segmental body composition for NHANES European-American normal women. Age is on the *x*-axis, and estimates of segmental body compositions from $M11_{B,E}\left(A\right)$ are on the *y*-axis. Reference Profile is represented by (o) and Gain Profile by (Δ).

Smooth curves are shown for normal EA men and women in Figure 1 and Figure 2**,** respectively. It is worth noting that the starting value at age 20 years in the RefProf context was higher than that in the GainProf context. Indeed, this is due to the effect of leverage associated with the lighter subject’s weight in other age intervals for the RefProf context. More precisely, given the same subset of subjects at 20–29 years in the RefProf and GainProf contexts, the RefProf parametric model fit is compromised due to high values at the left extremity of the curve. However, in the nonparametric modelling framework, the starting values at 20 years were the same in both RefProf and GainProf contexts.

In normal EA men, for the RefProf context, TF increased consistently with age from 20 years old, whereas BF was stable until age 40 and increased thereafter. Regarding lean masses, TL was more stable than APL and BL. Nevertheless, APL and BL declined from 20 years old. For the GainProf context, TF and BF generally increased at a constant rate. APL progressed and reached its maximum value at age 40–50 years and then declined slightly. Finally, TL and BL increased until 50 years, after which TL stabilised and BL declined.

In normal EA women, for the RefProf context, the results were similar to those of the men. For the GainProf context, TF and BF increased from 20 years, at a rate that was likely linear, whereas APL was nearly stable with age. The TL and BL values increased until 50 years. Then, TL stabilised and the BL declined slightly.

### 3.4. Profile Contexts with Effect of BMI and Ethnicity

#### 3.4.1. Reference Profile

As mentioned above, $M11_{B,\;E}\left(A\right)$ was retained to study BMI-, ethnicity- and age-related changes in body composition. Since the retained model was an additive model, the different BMI and ethnicity categories shared the same trends in body composition across age intervals, but with vertical linear translations. For the present study, the baselines for the BMI and ethnicity categories were “BMI = Normal” and “Ethnicity = EA”, respectively. The parameter differences between the other BMI and ethnicity categories and their corresponding baselines are summarised in Table 8.

In men, for the effect of ethnicity, AA men had lower TF, BF and TL masses than EA peers (differences of −1.48, −1.31 and −0.83 kg, respectively), had greater APL and BL masses (differences of 2.19 and 1.52 kg, respectively). For the effect of BMI, overweight and obese men always had greater SBCs than normal-weight men. The differences varied from 3.53 to 7.36 kg, respectively for APL and BL between the overweight and normal categories, whereas it varied from 5.49 to 11.77 kg between the obese and normal categories.

Similar results were found in women. AA women had −0.97, −0.58 and −0.6 kg lower TF, BF and TL, respectively, than EA peers, and had 1.9 and 1.55 kg greater APL and BL, respectively. Within the BMI categories, overweight women were 4.44 and 8.12 kg higher in BL and BF, respectively, than normal weight women, and these differences increased to 8.09 and 14.17 kg when comparing normal and obese women.

Regarding the effect of ethnicity compared to men, the absolute values of parameter differences in women were also greater in body and segmental fat masses, and lower in lean masses. With respect to the effect of BMI, the parameter differences in women were higher than in men for APL and BF masses, and were lower for APL, TL and BL masses.

#### 3.4.2. Gain Profile

The same procedure was applied to the study case of weight gain profile, using retained model $M11_{B,\;E}\left(A\right)$. Due to the properties of additive models, the BMI and ethnicity variables followed similar age-related trends in body composition. The parameter differences between other BMI and ethnicity categories and their baselines are shown in Table 8.

In both men and women, for the effects of BMI and ethnicity, similar results were observed as in the RefProf context: (1) AA subjects had lower TF, BF and TL than EA peers, but had greater APL and BL masses; (2) for the effect of BMI, overweight and obese subjects always had higher SBC than normal-weight subjects. Nevertheless, compared with the RefProf context, the parameter differences were greater in the GainProf context.

## 4. Discussion

The aim of this study was to assess age-related changes in body composition and to investigate how different BMI and ethnic categories affected this change during lifespan. By assuming two different secular trends of anthropometric covariates (RefProf and GainProf), our study showed a positive secular trend of TF and BF, irrespective of weight context and gender, a negative secular trend of body lean segments except TL for the RefProf concext in men and a U-shape trend in APL and BL for the GainProf context in men, whereas a nearly stable trend of APL, a positive secular trend of TL and a slight U-shape of BL for the GainProf context in women. In addition, our study demonstrated ethnic effect on change in SBC across the age intervals.

### 4.1. Age-Related Trends in Segmental Body Composition

Age-related changes in SBC are affected by a variety of factors such as physical activity, menopausal status, nutrition and disease [31]. Understanding these factors will help to assist in the prevention of functional limitation. In addition, establishing the nature of age-related changes will be useful for the health management of aging individuals. In theory, longitudinal studies are more reliable than cross-sectional studies of aging, but often have major drawbacks [32]. Recently, Ding et al. [18] used an approach that integrated cross-sectional and longitudinal studies. They found that the youngest cohorts had greater body fat mass than the oldest cohorts.

In the present study, we conducted a secular trend analysis with cross-sectional data. Primarily, we specified three BMI categories at 20 years with initial anthropometric values, and two representative weight-trend contexts (Reference and Gain Profiles) in aging within European- and African-American subjects. Then, we derived a nonparametric model to predict body composition changes in these various contexts, and a parametric model for better graphical representation and interpretation. In the reference profile context, total and segmental lean masses declined from age 20, while body and trunk fat masses increased consistently. Following Gain Profile context, total and segmental fat masses increased with age, whereas lean masses increased until 50 years and then decreased slightly thereafter. Our findings are consistent with similar studies.

The topic of body composition changes during aging has been widely discussed. In an observational study of healthy subjects aged from 0–80 years, Henche et al. [33] determined changes in total and regional fat masses and the percentage of certain lean masses. They showed that in both genders, BF increased until 70 years, then declined slightly. With respect to body segments, TF increased until 55 years in men and 70 years in women, and then stabilized in both genders. In another observational study by Welch and Sowers [34], who used DXA to assess women aged 18–94, BF increased gradually with age until 56 years and then decreased. Nevertheless, in our study, for both the Reference and Gain Profile contexts, we found that BF and TF increased consistently from 20 to 70 years. These findings contrast with those of Henche et al. [33] and Welch and Sowers [34], and may be due to decreases in weight after 40–60 years, whereas we assumed either constant or increasing weight.

In a predictive study by Chumlea et al. [35], estimated body fat-free masses increased until age 60 and 45 years in men and women, respectively, after which they declined. In addition, in men aged 35–81 years, Atlantis et al. [36] used DXA to show that, compared with the baseline age group (35–44 years), BL decreases with age. In another study conducted by Welch and Sowers [34], BL stabilized until 57 years and then decreased with age. In our study, the prediction for women confirmed Welch and Sowers’s findings about age-related changes in BL; however, the age at which BL declined was lower, approximately 50 years in the Gain Profile context and 20 years in the Reference Profile context. Furthermore, in the gain profile context, APL increased until 40–50 years in men and then decreased slightly; however, it was more inclined to be stable in women. The variable TL progressed consistently in both genders.

The magnitude of BFF change with age has been studied in small, longitudinal datasets. The results of Hughes et al. [37] and Kyle et al. [38] showed that the age-related longitudinal changes in BFF were −1.2 and −0.9 kg/decade for men and −0.1 and −0.4 kg/decade for women, respectively. The differences in BFF change rates might be due to sample differences. Indeed, Kyle et al. [38] studied a BIA-based dataset in age 20–73 years [38], whereas Hughes et al. studied a hydrodensitometry-based dataset of elderly men and women (initial age 60.7 ± 7.8 years) [37]. By approximating some non-linear curve to linear in the Reference Profile context, we found a rate of −0.8 and −0.5 kg/decade in BL (BL is closely related to BFF) for men and women, respectively. In addition, the results showed rates of ±0.8 and ±0.5 kg/decade in TF, and −0.8 and −0.5 kg/decade in APL for men and women, respectively, in the Reference Profile context. Regarding the Gain Profile context, the rate rose to 2 kg/decade in BF for both men and women.

To summarize, our results for BL change rates confirm Hughes et al.’s findings. As such, our methodology shows promise in being able to base secular trend analysis on cross-sectional data. In addition, rates of change in other body segments have been quantified. These findings are of interest from a physiological standpoint, especially when long-term longitudinal datasets are lacking.

### 4.2. Effect of Ethnicity

Ethnic differences in body composition have been reported in the USA [39]. The accumulation of fat masses, in particular trunk fat masses, is strongly related to age and ethnicity in both men and women [40]. Secondly, ethnicity-related differences in body composition may occur primarily in early adulthood [41]. In the present stud, we found that (1) ethnicity-related differences occur in SBC with aging; (2) based on the retained additive model, these differences are constant within each age interval. Moreover, our study suggests that across age intervals, African-American subjects had lower trunk fat, trunk lean and body fat masses than their European-American peers, and greater total and appendicular lean masses. Our findings are consistent with the previous conclusion there are ethnic differences in body composition and that they remain constant over the entire lifespan. Indeed, by using a DXA-based dataset from healthy Mexican-American and European-American women aged 20–75 years, Casas et al. showed that European-Americans may have modestly lower body and trunk fat masses and slightly higher body fat-free mass, particularly in the trunk region compared with Mexican-Americans [41]. Moreover, their findings showed that ethnicity-related differences in body composition were greatest in the young adults to the early-middle-aged age classes. Thus, they suggested that ethnicity-related differences may occur in early adulthood.

Some early studies have shown that ethnicity is an important factor in explaining the relationship between body fat and BMI [16]. Fernandez et al. [42] reported that the prediction of BF percentage based on the BMI of Mexican-American women differs from that of European- and African-American women. However, there were no significant differences between European- and African-American women, or between men of any ethnicities. Contrary to previous findings on BF percentage, we directly studied the amount of BF per kilogram. Furthermore, the present study demonstrates significant differences in BF and other SBC factors between European- and African-American subjects. Specifically, at the same BMI level, European-American subjects had lower body fat masses than African-American peers.

By studying an elderly cohort including Mexican-, European- and African-American subjects aged 60–98 years, Aleman-Mateo et al. [43] showed that, after controlling for BMI and age, there were ethnicity-related differences in body composition. African-Americans had lower body and trunk fat masses than their European-American peers, and greater total and appendicular lean masses. Our results support these findings with quantified difference values. Specifically, in the Gain Profile context, African-American subjects had about 2 and 1.5 kg greater APL and BL, respectively, whereas they were 1 kg lower in TF than their European-American peers. In another study using a BIA dataset and multicomponent model-derived prediction formulae, Chumlea et al. [35] found that the means for body fat-free masses within different ethnicities displayed similar patterns across age classes. Our retained additive model agrees with this finding. For the African-American category, age-related trends in body composition are transposed vertically compared with the model for European-American subjects.

### 4.3. Limitations

Several limitations of this study should be considered. First, the weight trend contexts were based on published findings; therefore, precision may be lacking. To ensure precision, an independent weight trend function could be developed for use in future studies. Secondly, this study used a cross-sectional dataset for a long-term trend analysis. Nonparametric modelling enabled us to extract a subset of similar subjects for a given age interval. However, it was unable to account for birth cohort effects such as the effect of height (previous generations are shorter than more recent generations, as height increases about 1 cm/decade). One potential way to adjust individual height is by correcting the data such that all subjects were born in the same year of subjects aged 20 years in the NHANES. This adjustment will be taken into account in the future study by using an independent height function with aging. Moreover, the additive model was used to describe overall long-term age-related changes rather than to accurately estimate body composition values. For further clinical use of this model, another validation study should be conducted.

## 5. Conclusions

In summary, we assessed age-related changes in segmental body composition and the influences of BMI and ethnicity. A nonparametric model was proposed to conduct a long-term trend analysis based on a cross-sectional dataset. Furthermore, we developed a parametric model for a smooth graphical presentation of age-related changes in body composition. Ethnic differences were found in body fat and lean masses, as well as in appendicular and trunk regions, findings which are consistent with previous studies. We provided additional quantitative information on ethnic differences, which we found to be constant across the lifetimes of adult humans.

## Figures and Tables

**Table 1:** Starting value of covariates (age, height, weight, waist circumference) for the three body mass index (BMI) categories at 20 years (BMIref) in men and women.

Gender		Normal (BMI = 22 kg/m^2^)	Overweight (BMI = 27 kg/m^2^)	Obese (BMI = 30 kg/m^2^)
		(Age, Height, Weight, Waist)	(Age, Height, Weight, Waist)	(Age, Height, Weight, Waist)
Gender	Men	(20 years, 175 cm, 67 kg, 85 cm)	(20 years, 175 cm, 85 kg, 95 cm)	(20 years, 175 cm, 95 kg, 105 cm)
	Women	(20 years, 165 cm, 60 kg, 83 cm)	(20 years, 165 cm, 75 kg, 95 cm)	(20 years, 165 cm, 85 kg, 105 cm)

**Table 2:** Schema of building 12 subsets associated with 12 analytic cases for nonparametric models and 2 subsamples for parametric models for a given gender.

Analytic Case Number	Weight Profile	Ethnic Group	BMI Category	Notation of Subset from Nonparametric Models	Notation of Subsample for Parametric Models
1	RefProf	European-American	Normal	subset_RefProf_#1	RefProf Subset
2	RefProf	European-American	Overweight	subset_RefProf_#2	
3	RefProf	European-American	Obese	subset_RefProf_#3	
4	RefProf	African-American	Normal	subset_RefProf_#4	
5	RefProf	African-American	Overweight	subset_RefProf_#5	
6	RefProf	African-American	Obese	subset_RefProf_#6	
7	GainProf	European-American	Normal	subset_GainProf_#1	GainProf Subset
8	GainProf	European-American	Overweight	subset_GainProf_#2	
9	GainProf	European-American	Obese	subset_GainProf_#3	
10	GainProf	African-American	Normal	subset_GainProf_#4	
11	GainProf	African-American	Overweight	subset_GainProf_#5	
12	GainProf	African-American	Obese	subset_GainProf_#6	

Note: RefProf: weight Reference Profile; GainProf: weight Gain Profile.

**Table 3:** Proposed parametric models for assessing the effect of BMI category and ethnicity, respectively, for a given gender and weight trend context.

Label	Model	Number of Free Parameters
Effect of BMI		
$M0_{B}\left(A\right)\;$*	$SBC=\;\eta +\;\alpha \times A+\beta \times A^{2}$	3
$M1_{B}\left(A\right)\;$*	$SBC=\;\eta_{B}+\;\alpha \times A+\beta \times A^{2}$	5
$M2_{B}\left(A\right)\;$*	$SBC=\eta_{B}+\;\alpha_{B}\times A+\beta \times A^{2}$	7
$M3_{B}\left(A\right)\;$*	$SBC=\eta_{B}+\;\alpha_{B}\times A+\beta_{B}\times A^{2}$	9
Effect of ethnicity		
$M10_{B,E}\left(A\right)\;$^†^	$SBC=\;\eta_{B}+\;\alpha \times A+\beta \times A^{2}$	5
$M11_{B,E}\left(A\right)\;$^†^	$SBC=\;\mu_{E}+\;\eta_{B}+\;\alpha \times A+\beta \times A^{2}$	7
$M12_{B,E}\left(A\right)\;$^†^	$SBC=\;\mu_{E}+\;\eta_{B,E}+\;\alpha \times A+\beta \times A^{2}$	11
$M13_{B,E}\left(A\right)\;$^†^	$SBC=\;\mu_{E}+\;\eta_{B,E}+\;\alpha_{E}\times A+\beta \times A^{2}$	13
$M14_{B,E}\left(A\right)\;$^†^	$SBC=\;\mu_{E}+\;\eta_{B,E}+\;\alpha_{E}\times A+\beta_{E}\times A^{2}$	15

Notes: BMI effect was examined first, followed by ethnicity. * $Mi_{B}\left(A\right)$ denotes models associated with the effect of BMI category. ^†^ $M1i_{B,E}\left(A\right)$ models are extensions of $M1_{B}\left(A\right)$, and model the effects of BMI and ethnicity.

**Table 4:** Men’s segmental body composition variables (mean ± standard deviation) obtained by DXA in National Health and Nutrition Examination Surveys (NHANES) and classified by age and ethnicity.

Ethnicity	Variables	20–29 years	30–39 years	40–49 years	50–59 years	60–69 years	>70 years
**European-American**	***n***	285	289	297	270	312	531
	**Height**	178.77 ± 6.98	177.76 ± 7.08	178.47 ± 6.70	177.26 ± 7.05	176.37 ± 6.71	172.37 ± 7.08
	**Weight**	84.62 ± 15.93	86.54 ± 16.34	89.54 ± 15.43	89.30 ± 14.95	90.18 ± 15.57	80.74 ± 14.09
	**Waist**	92.78 ± 12.61	96.35 ± 12.63	100.57 ± 11.54	103.15 ± 11.85	106.34 ± 12.09	102.38 ± 11.12
	**BMI**	26.45 ± 4.60	27.33 ± 4.56	28.06 ± 4.20	28.44 ± 4.61	28.94 ± 4.46	27.11 ± 4.05
	**BMI category (*n*)**						
	**Normal weight**	122	97	75	61	61	169
	**Overweight**	103	115	135	118	137	242
	**Obese**	60	77	87	91	114	120
	**TF**	9.08 ± 4.99	10.46 ± 5.12	11.90 ± 4.80	13.18 ± 5.23	14.33 ± 5.15	12.28 ± 4.49
	**BF**	19.03 ± 8.84	20.37 ± 8.49	22.27 ± 7.70	23.87 ± 8.31	25.43 ± 8.25	22.66 ± 7.39
	**APL**	28.52 ± 4.24	28.71 ± 4.58	28.80 ± 4.43	27.55 ± 3.95	26.87 ± 4.23	23.60 ± 3.76
	**TL**	30.73 ± 4.41	31.11 ± 4.64	32.09 ± 4.52	31.57 ± 4.17	31.62 ± 4.46	28.60 ± 4.15
	**BL**	62.83 ± 8.65	63.41 ± 9.25	64.49 ± 8.93	62.74 ± 8.10	62.08 ± 8.67	55.63 ± 7.96
**African-American**	***n***	130	131	150	98	117	95
	**Height**	177.57 ± 7.52	177.15 ± 7.19	176.81 ± 6.41	176.16 ± 6.98	175.22 ± 7.36	171.95 ± 7.08
	**Weight**	83.85 ± 17.65	85.01 ± 15.92	86.76 ± 16.20	87.84 ± 17.43	87.43 ± 15.86	80.97 ± 15.38
	**Waist**	87.73 ± 13.80	91.39 ± 12.10	95.36 ± 12.76	99.79 ± 13.57	101.34 ± 12.51	99.94 ± 11.54
	**BMI**	26.58 ± 5.34	27.03 ± 4.43	27.69 ± 4.58	28.20 ± 4.80	28.44 ± 4.62	27.31 ± 4.50
	**BMI category (*n*)**						
	**Normal weight**	63	45	52	25	27	31
	**Overweight**	33	53	53	39	48	43
	**Obese**	34	33	45	34	42	21
	**TF**	7.05 ± 4.72	8.11 ± 4.27	9.51 ± 4.65	10.70 ± 5.11	11.11 ± 5.13	11.12 ± 4.55
	**BF**	16.62 ± 9.46	17.43 ± 7.76	19.42 ± 8.07	20.82 ± 8.73	21.49 ± 8.47	21.88 ± 7.87
	**APL**	31.06 ± 5.39	30.97 ± 5.02	30.49 ± 5.02	29.71 ± 5.00	28.69 ± 4.33	25.38 ± 4.61
	**TL**	29.36 ± 4.76	29.93 ± 4.47	30.29 ± 4.56	30.68 ± 4.69	30.56 ± 4.55	27.46 ± 4.04
	**BL**	64.17 ± 10.27	64.57 ± 9.42	64.46 ± 9.59	64.15 ± 9.74	63.07 ± 8.82	56.47 ± 8.66

Notes: TF, trunk fat; BF, body fat; TL, trunk lean; APL, appendicular lean; BL, body lean.

**Table 5:** Women’s segmental body composition variables (mean ± standard deviation) obtained by DXA in NHANES and classified by age and ethnicity.

Ethnicity	Variables	20–29 years	30–39 years	40–49 years	50–59 years	60–69 years	>70 years
**European-American**	***n***	228	278	271	245	292	516
	**Height**	164.51 ± 5.84	164.24 ± 6.23	164.33 ± 6.53	163.09 ± 5.98	162.31 ± 6.09	158.03 ± 5.75
	**Weight**	68.48 ± 12.81	71.28 ± 15.00	74.33 ± 16.29	74.35 ± 13.55	74.29 ± 13.17	67.24 ± 12.23
	**Waist**	85.38 ± 12.10	87.67 ± 12.30	91.01 ± 13.57	93.82 ± 13.02	95.86 ± 12.38	94.72 ± 11.94
	**BMI**	25.32 ± 4.69	26.41 ± 5.33	27.48 ± 5.56	28.04 ± 5.39	28.19 ± 4.68	26.90 ± 4.48
	**BMI category (*n*)**						
	**Normal weight**	131	143	103	88	88	189
	**Overweight**	57	70	83	74	102	201
	**Obese**	40	65	85	83	102	126
	**TF**	10.14 ± 4.95	11.46 ± 5.52	12.83 ± 5.85	13.97 ± 5.24	14.67 ± 4.83	12.66 ± 4.36
	**BF**	23.27 ± 8.53	25.41 ± 9.80	27.50 ± 10.41	28.77 ± 8.86	29.90 ± 8.15	26.45 ± 7.81
	**APL**	18.39 ± 2.63	18.49 ± 3.08	18.70 ± 3.34	17.98 ± 2.82	17.46 ± 2.90	15.80 ± 2.56
	**TL**	21.72 ± 2.70	22.29 ± 3.10	22.98 ± 3.36	22.57 ± 3.13	22.01 ± 3.09	20.45 ± 2.79
	**BL**	43.05 ± 5.28	43.70 ± 6.18	44.63 ± 6.72	43.50 ± 5.90	42.42 ± 5.97	39.09 ± 5.30
**African-American**	***n***	107	130	157	89	121	93
	**Height**	162.90 ± 5.88	164.76 ± 6.77	162.94 ± 5.99	163.64 ± 7.28	162.86 ± 6.39	159.28 ± 5.69
	**Weight**	73.81 ± 15.71	78.74 ± 16.91	81.25 ± 14.38	79.38 ± 14.09	80.00 ± 15.17	74.32 ± 14.94
	**Waist**	88.35 ± 13.60	92.41 ± 13.33	97.13 ± 12.05	96.92 ± 12.38	99.11 ± 12.45	97.60 ± 12.36
	**BMI**	27.78 ± 5.59	28.93 ± 5.56	30.58 ± 5.00	29.68 ± 5.12	30.12 ± 5.21	29.18 ± 5.03
	**BMI category (*n*)**						
	**Normal weight**	40	36	24	20	21	21
	**Overweight**	32	39	46	34	42	31
	**Obese**	35	55	87	35	58	41
	**TF**	10.92 ± 5.60	12.60 ± 5.69	14.11 ± 4.76	14.41 ± 4.94	14.74 ± 5.03	12.99 ± 4.59
	**BF**	25.61 ± 10.16	28.26 ± 10.23	31.01 ± 8.90	31.29 ± 8.75	31.09 ± 9.57	28.63 ± 9.06
	**APL**	21.16 ± 3.49	22.04 ± 4.08	21.50 ± 3.28	20.12 ± 3.25	20.45 ± 3.50	18.99 ± 3.47
	**TL**	21.50 ± 3.00	22.73 ± 3.57	23.08 ± 3.01	22.50 ± 3.23	23.01 ± 3.20	21.61 ± 3.25
	**BL**	45.88 ± 6.52	48.05 ± 7.72	47.84 ± 6.21	45.83 ± 6.38	46.76 ± 6.66	43.75 ± 6.67

Notes: TF, trunk fat; BF, body fat; TL, trunk lean; APL, appendicular lean; BL, body lean.

**Table 6:** For effect of BMI, standard error of the estimate (kg) of different models in European-American men and women. In columns, the five segmental body compositions, in rows, the different models for two weight trend contexts.

Gender	Weight Change Context	Model	TF	BF	APL	TL	BL
Men	Reference Profile	Nonparametric	2.43	3.88	2.37	2.17	4.23
		$M0_{B}\left(A\right)$	3.54	5.74	3.09	3.09	6.05
		$M1_{B}\left(A\right)$	2.43	3.88	2.35	2.16	4.2
		$M2_{B}\left(A\right)$	2.43	3.88	2.35	2.16	4.2
		$M3_{B}\left(A\right)$	2.43	3.87	2.34	2.15	4.18
	Gain Profile	Nonparametric	2.51	3.97	2.46	2.26	4.39
		$M0_{B}\left(A\right)$	3.91	6.42	3.25	3.36	6.51
		$M1_{B}\left(A\right)$	2.52	3.99	2.45	2.29	4.42
		$M2_{B}\left(A\right)$	2.49	3.94	2.45	2.27	4.4
		$M3_{B}\left(A\right)$	2.48	3.91	2.45	2.27	4.4
Women	Reference Profile	Nonparametric	2.47	4.16	1.74	1.76	3.28
		$M0_{B}\left(A\right)$	3.96	6.91	2.26	2.36	4.52
		$M1_{B}\left(A\right)$	2.5	4.19	1.75	1.78	3.3
		$M2_{B}\left(A\right)$	2.49	4.19	1.75	1.78	3.3
		$M3_{B}\left(A\right)$	2.49	4.19	1.75	1.78	3.3
	Gain Profile	Nonparametric	2.57	4.38	1.75	1.79	3.32
		$M0_{B}\left(A\right)$	4.2	7.41	2.36	2.45	4.71
		$M1_{B}\left(A\right)$	2.59	4.44	1.76	1.81	3.34
		$M2_{B}\left(A\right)$	2.59	4.42	1.76	1.8	3.33
		$M3_{B}\left(A\right)$	2.59	4.42	1.76	1.8	3.33

Notes: TF, trunk fat; BF, body fat; TL, trunk lean; APL, appendicular lean; BL, body lean.

**Table 7:** For effect of ethnicity, standard error of the estimate (kg) of different models in men and women. In columns, the five segmental body compositions, in rows, the different models for two weight trend contexts.

Gender	Weight Change Context	Model	TF	BF	APL	TL	BL
Men	Reference Profile	Nonparametric	2.34	3.76	2.36	2.14	4.16
		$M10_{B,E}$	2.45	3.83	2.55	2.18	4.27
		$M11_{B,E}\left(A\right)$	2.35	3.78	2.34	2.14	4.15
		$M12_{B,E}\left(A\right)$	2.35	3.79	2.34	2.14	4.15
		$M13_{B,E}\left(A\right)$	2.34	3.78	2.34	2.14	4.15
		$M14_{B,E}\left(A\right)$	2.34	3.78	2.34	2.14	4.15
	Gain Profile	Nonparametric	2.43	3.88	2.43	2.23	4.32
		$M10_{B,E}\left(A\right)$	2.53	3.95	2.66	2.3	4.5
		$M11_{B,E}\left(A\right)$	2.44	3.91	2.44	2.27	4.38
		$M12_{B,E}\left(A\right)$	2.43	3.91	2.43	2.27	4.37
		$M13_{B,E}\left(A\right)$	2.43	3.91	2.44	2.27	4.37
		$M14_{B,E}\left(A\right)$	2.43	3.91	2.43	2.27	4.37
Women	Reference Profile	Nonparametric	2.41	4.16	1.79	1.74	3.29
		$M10_{B,E}\left(A\right)$	2.52	4.22	1.98	1.78	3.41
		$M11_{B,E}\left(A\right)$	2.44	4.2	1.8	1.76	3.32
		$M12_{B,E}\left(A\right)$	2.44	4.2	1.8	1.76	3.32
		$M13_{B,E}\left(A\right)$	2.44	4.2	1.8	1.76	3.31
		$M14_{B,E}\left(A\right)$	2.44	4.2	1.8	1.76	3.31
	Gain Profile	Nonparametric	2.51	4.36	1.8	1.78	3.32
		$M10_{B,E}\left(A\right)$	2.61	4.41	2.02	1.83	3.47
		$M11_{B,E}\left(A\right)$	2.53	4.4	1.82	1.81	3.37
		$M12_{B,E}\left(A\right)$	2.53	4.4	1.82	1.81	3.37
		$M13_{B,E}\left(A\right)$	2.53	4.4	1.82	1.8	3.36
		$M14_{B,E}\left(A\right)$	2.53	4.4	1.81	1.8	3.36

Notes: TF, trunk fat; BF, body fat; TL, trunk lean; APL, appendicular lean; BL, body lean.

**Table 8:** Parameter differences of the ethnicity and BMI categories from their baselines, which were “Ethnicity = EA” and “BMI = normal weight”, respectively. Values are given for men and women, according to the retained model $M11_{B,E}\left(A\right)$.

Gender	Weight Change Context	Parameter Differences	TF	BF	APL	TL	BL
Men	Reference Profile	$\mu_{AA}-\mu_{EA}$	−1.48	−1.31	2.19	−0.83	1.52
		$\eta_{OW}-\eta_{N}$	3.8	6.37	3.53	3.57	7.36
		$\eta_{OB}-\eta_{N}$	6.87	11.54	5.49	5.87	11.77
	Gain Profile	$\mu_{AA}-\mu_{EA}$	−1.34	−1.06	2.36	−0.76	1.78
		$\eta_{OW}-\eta_{N}$	4.45	7.67	3.63	3.89	7.8
		$\eta_{OB}-\eta_{N}$	7.67	13.02	5.76	6.39	12.6
Women	Reference Profile	$\mu_{AA}-\mu_{EA}$	−0.97	−0.58	1.9	−0.6	1.55
		$\eta_{OW}-\eta_{N}$	4.46	8.12	2.04	2.23	4.44
		$\eta_{OB}-\eta_{N}$	7.78	14.17	3.8	4.01	8.09
	Gain Profile	$\mu_{AA}-\mu_{EA}$	−0.96	−0.44	2.01	−0.65	1.63
		$\eta_{OW}-\eta_{N}$	4.68	8.73	2.38	2.47	5.01
		$\eta_{OB}-\eta_{N}$	8.32	15.14	4.17	4.27	8.72

Notes: TF, trunk fat; BF, body fat; TL, trunk lean; APL, appendicular lean; BL, body lean; AA, African-American; EA, European-American; OW, overweight; N, normal weight; OB, obesity.

## Abbreviations

The following abbreviations are used in this manuscript:

BL	Body lean masses
BFF	Body fat-free masses
BF	Body fat
SBC	Segmental body composition
DXA	Dual-energy X-ray absorptiometry
NHANES	National Health and Nutrition Examination Survey
EA	European-American
AA	African-American
TF	Trunk fat masses
TL	Trunk lean masses
APL	Appendicular lean masses
BMIRef	BMI at the age of 20 years
RefProf	Reference profile
GainProf	Gain profile
SEE	Standard error of the estimate
